# Longitudinal Cochlear Implant Outcomes in Danish Adults: Changes in Speech Recognition, Self-Reported Hearing Ability, Hearing-Related Quality of Life, and Tinnitus

**DOI:** 10.3390/jcm14176124

**Published:** 2025-08-29

**Authors:** Line Husted Baungaard, Matilde Grønborg Sandvej, Mathilde Marie Overmark Nellemose, Marianne Kyhne Hestbæk, Louise Brasen Brændgaard, Mie Stenner Hansen, Mie Lærkegård Jørgensen, Per Cayé-Thomasen, Lone Percy-Smith

**Affiliations:** 1 Copenhagen Hearing and Balance Centre, Ear, Nose and Throat (ENT) and Audiology Clinic, Rigshospitalet, Inge Lehmanns Vej 8, 2100 Copenhagen, Denmark; matilde.groenborg.sandvej@regionh.dk (M.G.S.); mathilde.marie.overmark@regionh.dk (M.M.O.N.); marianne.kyhne.hestbaek@regionh.dk (M.K.H.); louise.brasen.braendgaard@regionh.dk (L.B.B.); mie.stenner.hansen@regionh.dk (M.S.H.); per.caye-thomasen@regionh.dk (P.C.-T.); lone.percy-smith@regionh.dk (L.P.-S.); 2 Hearing Systems, Department of Health Technology, Technical University of Denmark, 2800 Kongens Lyngby, Denmark; mielj@dtu.dk

**Keywords:** adults, adaptive hearing in noise test (HINT), cochlear implant (CI), hearing-related quality of life (HR-QOL), longitudinal outcomes, patient-reported outcome measures (PROMs), speech recognition

## Abstract

**Background:** This study assessed the efficacy and effectiveness of cochlear implants (CIs) by examining changes in speech recognition, self-reported hearing ability, hearing-related quality of life, and tinnitus over two years. **Methods:** A prospective two-year longitudinal study included 50 adult users of CI. Speech recognition in quiet and background noise was measured using Dantale I (with/without visual cues; fixed 0 dB SNR) and the adaptive Hearing in Noise Test (HINT). Self-reported outcomes were obtained using the Speech, Spatial, and Qualities of Hearing Scale (SSQ-12), Nijmegen Cochlear Implant Questionnaire (NCIQ), and Tinnitus Handicap Inventory (THI). **Results:** In post-lingually deafened users of CI (N=46), Dantale with visual cues improved from 74.2% to 94.9% (mean difference = 20.7% [95% CI: 15.8, 25.6], p<0.001, $d_{z}=1.83$), and without visual cues from 58.9% to 92.1% (33.1% [28.0, 38.2], p<0.001, $d_{z}=2.83$). Dantale with background noise improved from 23.4% to 61.7% (37.6% [32.4, 42.8], p<0.001, $d_{z}=3.13$), and adaptive HINT from 8.6 to 4.2 dB SNR (−5.8 [−7.0, −4.6], p<0.001, $d_{z}=3.10$). NCIQ scores increased from 292.6 to 436.3 points (145.3 [122.8, 167.8], p<0.001, $d_{z}=3.11$), and SSQ-12 improved by 0.60 points [0.18, 1.02] from 1 to 2 years (p=0.015, $d_{z}=0.86$). Tinnitus severity remained low at the group level, with individual fluctuations over time. **Conclusions:** Danish adult users of CI showed substantial and sustained improvements in speech recognition and hearing-related quality of life over two years, with adaptive speech-in-noise testing proving particularly sensitive for detecting long-term changes.

## 1. Introduction

Worldwide, 430 million people—equivalent to 1 in every 20 individuals—live with a disabling hearing loss. The World Health Organization (WHO) defines disabling hearing loss as a hearing loss greater than 35 dB in the better ear. By 2050, this number is projected to rise to nearly 700 million people, or 1 in every 10 individuals [1]. When living with hearing loss, communication and everyday listening—especially in the presence of background noise—typically become more effortful. This increased listening effort requires greater attention and more top–down language processing to compensate for the reduced auditory input. As a result, hearing loss can negatively affect a person’s cognitive functioning, as well as their social and personal well-being [2,3,4,5,6].

Hearing technology, such as hearing aids (HAs) or bone-anchored HAs, can be effective in treating mild to moderate sensorineural or conductive hearing loss. For individuals with congenital or acquired severe-to-profound sensorineural hearing loss, a cochlear implant (CI) may be indicated. CIs can be used in different configurations depending on the individual’s binaural hearing status: unilateral configuration (CI use in one ear with no hearing technology use in the other), bimodal configuration (CI use in one ear and HA use in the other), or binaural configuration (CI use in both ears). Hearing technology aims to provide access to the full range of surrounding sounds, including both speech and environmental sounds. The long-term safety, reliability, efficacy, effectiveness, and cost-effectiveness of treatment with CI for severe-to-profound sensorineural hearing loss have been widely studied and well established [2,7,8,9,10,11]. In addition, person- and family-centred support for the user of hearing technology is an essential part of the treatment of hearing loss to maximise the auditory development after the fitting of the hearing technology [12].

When documenting the efficacy and effectiveness of CI treatment, Boisvert et al. (2020) emphasise the importance of assessing speech recognition ability as a measure of efficacy and self-reported outcomes as measures of effectiveness [7]. Assessing speech recognition requires careful consideration to accommodate the individual’s hearing and language abilities both before and after implantation. To minimise ceiling and floor effects, adaptive testing procedures have been developed and are somewhat applied in clinical practice. In research, the procedures are primarily used in studies focused on populations with binaural or bimodal CI configurations [7,13,14,15,16,17,18,19].

Comparing CI outcome results across regions and languages is a common clinical practice aimed at supporting counselling, managing expectations and assessing expected outcomes for each user of CI. Although numerous studies have reported on CI outcomes over the years, direct comparisons remain difficult due to substantial variations in study designs. Differences in participant characteristics, hearing technologies, the translations and cultural modifications of test materials, and follow-up timelines all contribute to this complexity [13,20,21,22,23,24,25]. In a systematic review and meta-analysis from 2023, Ma et al. included 22 longitudinal CI outcome studies, representing results from a total of 1954 users of CI [26]. Covering a selection of speech recognition tests, they report mean scores and mean differences of percentages correct for three types of tests—words in quiet, sentences in quiet, and sentences in background noise at five time points (before using a CI and at 3, 6, 12, and 24 months after the cochlear implantation).

In Denmark, no official national clinical guidelines currently exist for CI treatment and aftercare, including the assessment of efficacy and effectiveness. This study contributes to recent research on CI outcomes in adults by investigating a clinical representative population using an up-to-date clinical protocol. The aim was to evaluate long-term CI outcomes in a clinical setting by examining longitudinal changes in speech recognition, self-reported hearing ability, hearing-related quality of life, and tinnitus. All assessments were conducted while participants used their hearing technology as worn in everyday life. As part of the speech recognition assessment, an adaptive sentence-in-noise procedure was used to evaluate speech recognition in background noise.

## 2. Materials and Methods

The study used a longitudinal methodological design and took place at the CI centre at the Copenhagen Hearing and Balance Centre. Adult users of CI were invited to participate during their routine 12-month follow-up visit that was part of the standard clinical care. During the inclusion period, which spanned from December 2020 to January 2022, a total of 56 users of CI were invited to participate. Participants were included if they had had 10 to 15 months of CI use with their latest CI at the follow-up visit. Other inclusion criteria were fluency in Danish and the ability to participate in test activities without assistance. Two individuals were not included due to limited CI use—one as a result of other health-related issues, and the other due to changes in CI electrode impedance, which restricted mapping options throughout the inclusion period. A third individual experienced prolonged adaptation to the CI following postponed follow-up visits at the CI centre during the COVID-19 pandemic and was therefore not included. Of the 53 eligible users of CI who were informed about the study and invited to participate, three declined, leaving 50 who consented to participate. All participants signed an informed consent form, of which none withdrew during or after the study period. As illustrated in Table 1, participants were subsequently invited to attend follow-up visits at 18 and 24 months after the routine 12-month visit, during which additional information and data were collected prospectively.

### 2.1. Test Battery

To evaluate the main outcomes of cochlear implantation over time, we used measures of speech recognition, self-reported hearing ability, hearing-related quality of life, and tinnitus severity. Speech recognition in quiet was assessed using the Dantale I—or Dantale—test with and without visual cues. The Dantale test consisted of eight lists of 20 Danish monosyllabic words that were presented at a sound pressure level (SPL) of 65 dB SPL. Scores were recorded as the percentage of correct phonemes identified (Elberling et al., 1989) [27]. For the assessment of the speech recognition in background noise, two tests without visual cues were used: the one-word Dantale test and the sentence-based Hearing in Noise Test (HINT) with an adaptive procedure. The standard adaptive procedure was used for the HINT test, where the noise signal was fixed at 65 dB SPL and the speech level varied based on the accuracy of previously heard words within the sentence (Nielsen & Dau, 2011) [28]. The HINT Speech Reception Threshold (SRT) was recorded as a signal-to-noise ratio (SNR). As for the testing with Dantale in background noise, both the speech signal and the noise signal were presented at 65 dB SPL (the signal-to-noise ratio was 0 dB SNR), and the scores were recorded as the percentage of correct phonemes identified. The speech recognition testing was performed by trained clinicians in a sound booth, which was set up for clinical free-field speech testing.

Two questionnaires—that are widely used in clinical practice and research—were used to assess self-reported hearing ability and hearing-related quality of life: the Nijmegen Cochlear Implant Questionnaire (NCIQ) and the 12-item Speech, Spatial, and Qualities of Hearing Scale (SSQ-12). Both questionnaires were administered in culturally modified Danish translations [29,30,31]. The NCIQ was administered at baseline and at three follow-up time points after cochlear implantation. The SSQ-12 was introduced as a measure of self-reported hearing ability at study inclusion (at the 1-year follow-up), and therefore administered only at the three post-implant time points, with no baseline measurement, allowing for post-CI comparisons only. Another questionnaire was used for measuring change in tinnitus severity over time: the Tinnitus Handicap Inventory (THI) questionnaire [32].

All outcome measures were completed while the participants were wearing their hearing devices as they do in everyday life—reflecting typical listening conditions.

Background information about the participants’ gender, age, hearing history, duration of functional deafness, baseline hearing thresholds, and hearing technology configuration was collected from the medical journal at the time of inclusion.

### 2.2. Participant Characteristics

Table 2 summarises information about the participants’ gender, age, hearing history, and pre-implant hearing.

The group consisted of 25 female and 25 male participants with an age range from 37 to 83 years. The average age was 66.8 years. Most participants had post-lingual deafness (46 participants), two had pre-lingual deafness, and two had single-sided deafness. Participants reported having experienced functional deafness for between 1 and 20 years, with a mean duration of 3.8 years.

The pre-implant hearing loss in the CI ear and in the contralateral ear is given as the pure-tone average for the frequencies 250, 500, 1000, 2000, 4000, and 8000 Hz (PTA6). The number of ears was between 45 and 46. In cases of measured deafness prior to inclusion in this study, the hearing loss was not measured again and was, therefore, not noted.

In addition to the demographic information shown in Table 2, the vocational status of the participant group was that 34 were retired, 14 were employed (nine full-time and five part-time), and two were unemployed. As to their household status, 41 of the participants lived in a household with others.

At the time of inclusion, 42 participants had used their first CI for one year, and eight participants had used their second CI for one year. Among the 42 participants, three used a unilateral CI, 35 used a bimodal configuration, and four had recently received a second CI. In total, 12 participants used a binaural configuration at inclusion. In the subgroup of users of CI with post-lingual deafness, 32 used a bimodal configuration, 11 used a binaural configuration, and three used a unilateral CI configuration.

The majority of participants used a Cochlear CI device (37 participants), nine used an Advanced Bionics CI device, and four used an Oticon Medical CI device.

### 2.3. Statistical Methods

All analyses, illustrations, and summary statistics were conducted in RStudio v.2024.12.1. The statistical analyses include data from the group of 46 participants with post-lingual deafness. Due to the low number of participants with SSD (N = 2) and pre-lingual deafness (N = 2), the results for these participants are described, but not included, in the illustrations or the statistical analyses.

#### 2.3.1. Analyses of Efficacy and Effectiveness Measures

Changes in scores over time in speech recognition (Dantale and adaptive HINT), self-reported hearing ability (SSQ-12), and hearing-related quality of life (NCIQ) were evaluated using linear mixed-effects models (LMMs), adjusted for baseline values. Time was included as a fixed effect, and subject-level variability was modelled with random effects. Model-based variance was assessed via ANOVA. Post-hoc pairwise comparisons across time points were performed with the Holm correction for multiple testing. For all comparisons, standardised within-subject effect sizes were calculated by dividing the estimated marginal mean differences by the model’s residual standard deviation.

##### Assessment and Handling of Missing Data

To assess whether the LMMs could validly assume data were Missing At Random (MAR), response rates were examined by measure and time point (Appendix A, Table A1). Mixed-effects logistic regression models were fitted to predict a binary missingness indicator (1 = missing, 0 = observed) based on time point (baseline, 1, 1.5, and 2 years) and participants’ baseline score, with a random intercept for participant. Across speech perception measures (Dantale and HINT tests) and self-reported outcome measures (NCIQ and SSQ-12), the time point was a significant predictor of missingness, whereas baseline performance was not. In all cases, missingness was linked only to observed variables (time point) and not to unobserved outcome values, consistent with a MAR mechanism. Under this assumption, our likelihood-based LMMs yield unbiased estimates without requiring additional imputation or sensitivity analyses.

### 2.4. Analysis of Tinnitus Measures

THI scores were classified into six ordered categories according to standard severity levels. Changes in the distribution of participants across these categories over time were assessed using the Friedman test.

### 2.5. Use of GenAI in Writing

During the preparation of this manuscript, the authors utilised ChatGPT-4o and ChatGPT o4-mini-high (OpenAI) to support the editing of R code and to enhance the clarity and grammar of the written text. All AI-assisted outputs have been critically reviewed and revised by the authors, who take full responsibility for the final content of this publication.

## 3. Results

### 3.1. Efficacy Results for Participants with Post-Lingual Deafness

**Figure 1:**
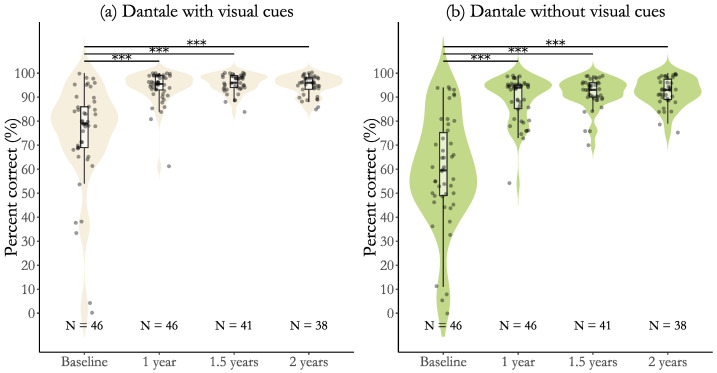
Speech understanding scores over time measured by the Dantale test with and without visual cues. Panel (**a**) shows results for the Dantale test with visual cues, and panel (**b**) without visual cues. Violin plots display the distribution of individual scores at each time point (baseline, 1 year, 1.5 years, and 2 years), overlaid with boxplots and individual data points. Sample sizes (N) are shown below each plot. Asterisks indicate statistically significant pairwise comparisons (*** = *p* < 0.001).

**Figure 2:**
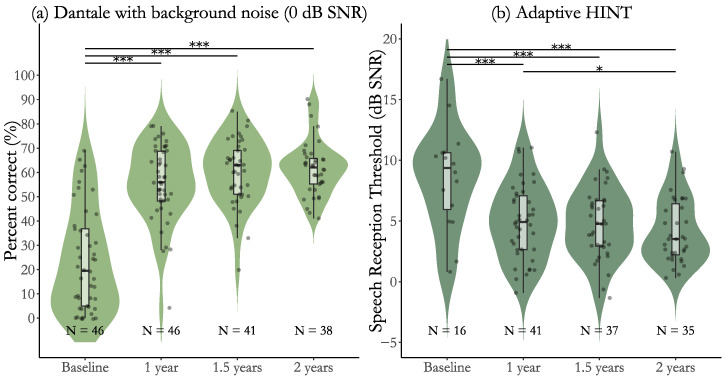
Speech understanding in noise across time points, measured by the Dantale test and adaptive HINT. Panel (**a**) shows results from the Dantale test presented in 0 dB SNR (without visual cues), and panel (**b**) displays results from the adaptive HINT test. Violin plots represent the distribution of individual scores across time points (baseline, 1 year, 1.5 years, 2 years), with overlaid boxplots and individual data points. Percentage correct scores are shown for Dantale, while HINT scores are measured in dB SNR (lower values indicate better performance). Sample sizes (N) are shown beneath each group. Asterisks indicate significant differences between time points (* *p* < 0.05, *** *p* < 0.001).

The speech recognition results for users of CI with post-lingual deafness are presented as violin plots in Figure 1 and Figure 2. Each violin plot includes an embedded box plot representing group scores, with individual data points overlaid as dots. The number of individual observations (N) is shown below each plot. Horizontal significance lines indicate time points with statistically significant changes in group scores. Figure 1 displays results from the one-word Dantale test with visual cues (a) and without visual cues (b). Figure 2 shows results from the Dantale test with background noise (without visual cues) (a) and from the adaptive HINT sentence test (b). Table 3 and Table 4 present the changes in group scores across time points, reported as estimated mean differences with standard errors and 95% confidence intervals, along with the Holm-adjusted *p*-value for each comparison. Cohen’s $d_{z}$ effect sizes with 95% confidence intervals are shown for each of the comparisons. Table 5 presents the means and standard deviations for all measures at each time point.

Overall, speech recognition scores improved significantly from baseline to 1 year, 1.5 years, and 2 years. The *Dantale with visual cues* scores (Figure 1 and Table 3) improved significantly from baseline to 1 year (19.8±2.36%, p<0.001), 1.5 years (21.5±2.44%, p<0.001), and 2 years (20.7±2.50%, p<0.001). These comparisons were associated with large effect sizes (Cohen’s $d_{z}>1.2$), indicating substantial improvement. Ceiling effects were evident at all time points after baseline. Post-hoc comparisons between time points were not significant and were associated with negligible effect sizes (Cohen’s $d_{z}<0.2$). The *Dantale without visual cues* scores (Figure 1 and Table 3) improved significantly from baseline to 1 year (30.3±2.44%, (p<0.001)), 1.5 years (32.7±2.53%, (p<0.001)), and 2 years (33.1±2.59%, (p<0.001)). All comparisons from baseline to after baseline were associated with large effect sizes (Cohen’s $d_{z}>1.2$). Ceiling effects were evident at all time points after baseline. Post-hoc comparisons between follow-ups were not significant, with negligible or very small effect sizes (Cohen’s $d_{z}\leq 0.2$) with 95% confidence intervals including zero.

The *Dantale with background noise* scores (Figure 2a and Table 4) improved significantly from baseline to 1 year (32.2±2.50%, (p<0.001)), 1.5 years (36.9±2.60%, (p<0.001)), and 2 years (37.6±2.67%, (p<0.001)). Large effect sizes were observed for all baseline-to-follow-up comparisons (Cohen’s $d_{z}>1.2$). The comparisons after baseline showed no evidence of ceiling effects. Small, non-significant improvements in speech recognition were observed, with small-to-medium effect sizes and 95% confidence intervals whose lower bounds were at or included zero.

The *adaptive HINT* scores (Figure 2b and Table 4) improved significantly from baseline to 1 year (−4.7dBSNR±0.59, (p<0.001)), 1.5 years (−5.1dBSNR±0.61, (p<0.001)), and 2 years (−5.8dBSNR±0.63, (p<0.001)). These improvements were associated with large effect sizes (Cohen’s $d_{z}>1.2$). Between time points after baseline, a small but significant improvement was observed from 1 to 2 years (−1.1dBSNR±0.45, (p=0.042)), corresponding to a medium effect size (Cohen’s $d_{z}=0.60$ [0.12, 1.09]). Other contrasts after baseline (1 year to 1.5 years; 1.5 to 2 years) were not significant and showed small-to-medium effect sizes with 95% confidence intervals including zero. No ceiling effects were observed for either the Dantale in noise or the adaptive HINT. For the adaptive HINT, however, the limited number of participants with measurable scores at baseline indicates the presence of a floor effect for this measure.

### 3.2. Efficacy Results for Participants with Pre-Lingual Deafness and SSD

Results were also recorded for two participants with pre-lingual deafness. At baseline, Dantale test scores ranged from 24% to 51% correct in the condition with visual cues, and from 0% to 39% without visual cues. In the background noise condition, scores ranged from 0% to 9%. At the one-year follow-up, scores improved to 38–89% with visual cues, 1–58% without visual cues, and 0–21% in background noise. At 1.5 years, scores with visual cues ranged from 44% to 88%; one participant scored 59% without visual cues and 18% in background noise. By the two-year follow-up, scores with visual cues ranged from 60% to 68%, while scores without visual cues ranged from 15% to 73%. Finally, one participant scored 13% correct in background noise.

Dantale results were also recorded for two participants with single-sided deafness (SSD). One participant completed assessments at baseline, one year, and two years, while the other participated at baseline and one year. Scores with and without visual cues ranged from 98% to 100% correct across all available time points. At baseline, background noise scores ranged from 60% to 88% increasing to 78% to 85% at one year. At two years, the background noise score declined to 50%. HINT results were likewise collected. At one year, scores ranged from 0.31 to −1.15 dB SNR. At two years, a decrease in performance was observed, consistent with the Dantale background noise results, as the HINT score had declined to 1.01 dB SNR.

### 3.3. Effectiveness for Participants with Post-Lingual Deafness

**Figure 3:**
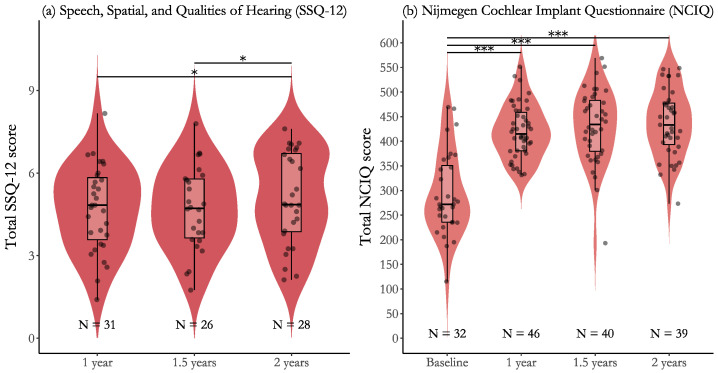
Subjective ratings of hearing ability, measured by SSQ-12, and hearing-related quality of life, measured by NCIQ. Panel (**a**) presents scores from the SSQ-12 across 1 year, 1.5 years, and 2 years after baseline. Panel (**b**) shows results from the NCIQ, including a baseline measure. Violin plots represent the distribution of individual scores, overlaid with boxplots and individual data points. Sample sizes (N) are shown beneath each group. Asterisks indicate significant differences between time points (* *p* < 0.05, *** *p* < 0.001). Higher scores indicate better perceived hearing outcomes.

The results from the 12-item Speech, Spatial, and Qualities (SSQ-12) and Nijmegen Cochlear Implant Questionnaire (NCIQ) questionnaires are shown in Figure 3 and Table 6. The *NCIQ* scores showed a large improvement in self-reported hearing-related quality of life from baseline to 1 year (130.5±11.1 points, (p<0.001)), 1.5 years (139.7±11.5 points, (p<0.001)), and 2 years (145.3±11.5 points, (p<0.001)). The corresponding effect sizes were substantial: $d_{z}=2.79$ (95% CI [2.22, 3.36]), $d_{z}=2.99$ (95% CI [2.39, 3.58]), and $d_{z}=3.11$ (95% CI [2.50, 3.71]). No statistically significant differences were observed between post-baseline time points, indicating that the major gains occurred within the first year after implantation and then remained stable. Effect sizes for post-baseline comparisons ranged from small to medium, with all 95% confidence intervals including zero.

The mean *SSQ-12* results do not allow for comparisons with baseline scores but reflect changes in self-reported hearing ability from year 1 onward. From year 1 to 2, scores increased by 0.60±0.21 points, (p=0.015), corresponding to an effect size of $d_{z}=0.86$ (95% CI [0.03, 1.05]). From year 1.5 to 2, the increase was 0.49±0.20 points, (p=0.042), with an effect size of $d_{z}=0.70$ (95% CI [0.10, 1.29]). In both cases, the lower limits of the 95% confidence intervals were close to zero. The comparison between year 1 and 1.5 years was not significant and had a negligible effect size.

### 3.4. Effectiveness for Participants with Pre-Lingual Deafness and SSD

The results of self-reported outcome measures were also collected for two participants with pre-lingual deafness. The mean scores for SSQ-12 at one year ranged from 4.25 to 4.88 points, increasing to 4.63 to 6.25 points at 1.5 years. At two years, one participant scored 4.67 points. The NCIQ total score was available for one participant at baseline, with a score of 288.9 points. At one year, scores ranged from 377.5 to 451.1 points; at 1.5 years, from 387.5 to 466.7 points; and at two years, from 366.4 to 474.4 points.

For the participants with single-sided deafness (SSD), SSQ-12 mean scores were available for one participant who scored 4.17 points at one year, increasing to 5.50 points at two years. NCIQ total scores at baseline ranged from 260.8 to 278.3 points for the two participants. One participant showed an increase at one year (435.3 points), followed by a decrease to 376.7 points at two years.

### 3.5. Tinnitus Results for Participants with Post-Lingual Deafness

**Figure 4:**
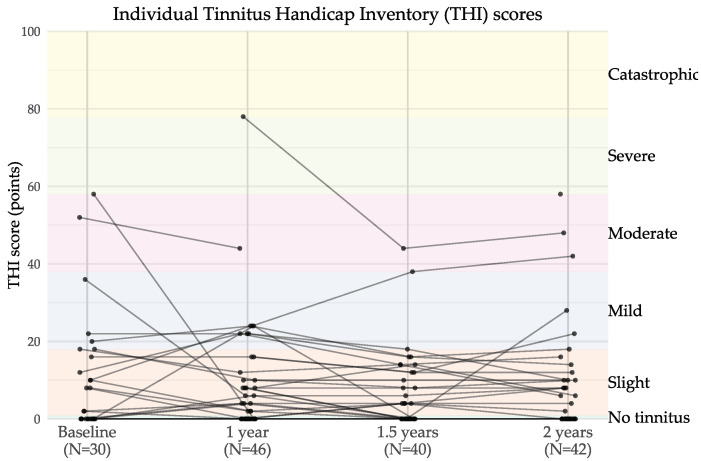
Individual THI scores over time for users of CI with post-lingual deafness. Each line represents one participant across time points. Background band colours indicate tinnitus severity categories.

Figure 4 presents the individual results from the *Tinnitus Handicap Inventory (THI)* across the four time points, and Table 7 shows the percentage of participants in each scoring category. The majority of participants had scores indicating no or slight tinnitus at all time points: baseline (50.0%), 1 year (82.7%), 1.5 years (80.4%), and 2 years (78.3%). There was no significant difference in tinnitus severity across time points (Friedman’s $\chi^{2}\left(3\right)=4.17$, p=0.24). However, some participants scored higher at later time points, indicating that tinnitus severity can fluctuate over time, both improving and worsening between assessments.

### 3.6. Tinnitus Results for Participants with Pre-Lingual Deafness and SSD

Results were also recorded for two participants with pre-lingual deafness. Total THI scores were not available at baseline. At one year, scores ranged from 0 to 40 points; at 1.5 years, from 0 to 34 points; and at two years, from 0 to 42 points. One participant with single-sided deafness (SSD) scored 58 points at baseline, showed a decrease to 22 points at one year, followed by an increase to 54 points at two years on the THI.

## 4. Discussion

### 4.1. Participant Characteristics

In this study, the participant group was comprised of a heterogeneous group in terms of hearing history and hearing technology configuration. The participants were included from a random clinical population over a predefined time period. The participant sample included a high proportion of *users of CI with post-lingual deafness* (N = 46), most of whom used a *bimodal configuration* (N = 32), resulting in over-representation of this subgroup in the sample. This likely influenced the overall outcome patterns and should be taken into account when interpreting the findings. The participant group used a variety of hearing technology configurations and CI brands, and variations in hearing outcomes between technology groups might be expected, as documented in previous research [20,33]. However, we did not stratify the post-lingual group by specific hearing technology, as the distribution across groups was unbalanced. The combination of small subgroup sizes and missing data precluded meaningful subgroup analyses.

Results for participants with pre-lingual deafness and single-sided deafness (SSD) were reported descriptively, but not included in the linear mixed-effects models due to the small number of cases. Future studies with larger subgroups are needed to enable more precise insights into the change in hearing ability and hearing-related quality of life after cochlear implantation in these populations.

### 4.2. Efficacy Results

Overall, participants with post-lingual deafness showed stable, clinically meaningful gains in speech recognition from baseline to 1, 1.5, and 2 years after baseline. Significant improvements, along with corresponding effect sizes, were observed for both the Dantale and adaptive HINT measures when comparing baseline with each of the three time points after baseline. The results were higher than the long-term outcomes reported in other studies that have used speech recognition as a measure of efficacy. Ma et al. [26] reported that word recognition scores in quiet for users of CI were 9.8% at baseline, increasing to 60.0% at one year and 60.3% at two years. Similarly, Cusumano et al. [13] found that among CI users with a bimodal configuration, word scores in quiet were 33.1% at baseline, 66.5% at one year, and 68.7% at two years. The higher baseline scores observed in the current study, compared to those reported by Ma et al. and Cusumano et al., suggest that the participant groups may have differed in hearing technology configuration during testing or in their pre-implant hearing histories—both of which could contribute to the observed differences in outcome trajectories. When comparing the current Dantale results at one year with best-aided one-year Dantale scores reported by Rasmussen et al. [21], the present word scores—both in quiet and in background noise—are also slightly higher. This difference may reflect variation in participant characteristics, as the present study also includes users of binaural CI in the sample.

The near-ceiling percentage correct scores and minimal effect sizes observed in the Dantale conditions in quiet after baseline, suggest that possible further improvements were limited by ceiling effects. When hearing ability was assessed at baseline, floor effects were observed when using the adaptive HINT measure. These effects limit the ability to capture meaningful, individual changes in speech perception before and after cochlear implantation, both in a clinical setting and in research. As a result, patients may appear to reach maximum or minimum scores, which can mask true variability and lead to misleading conclusions about hearing outcomes after cochlear implantation. This highlights the ongoing need for more sensitive and appropriately targeted speech perception materials that can track hearing changes both pre- and post-implantation, particularly at the individual level.

The adaptive HINT measure appeared sensitive to longitudinal changes in speech reception at time points after baseline that were not seen in the Dantale measure in background noise. As the Dantale in the background noise condition was administered at a fixed SNR of 0 dB, it may lack the sensitivity to detect subtle, long-term improvements in hearing ability. This observation supports that the use of an adaptive procedure when testing speech recognition in background noise is a more sensitive and informative tool for monitoring long-term improvements in hearing ability compared to measures that use a static SNR level in background noise [13,14]. The adaptive HINT test appears to offer promise in this regard.

### 4.3. Effectiveness Results

The self-reported outcome NCIQ results seen in this study also showed that participants with post-lingual deafness experienced significant, stable, and clinically meaningful improvements over time. Although there is no consensus on what constitutes a clinically relevant difference for the NCIQ, comparing the current results with those of similar studies can still provide valuable insight into how different users of CI score on these measures [34]. The current results are comparable or higher to other CI outcome studies that use NCIQ as a measure for effectiveness [21,23,24,25]. The current NCIQ results are comparable to the total scores reported by Hirschfelder et al. [24] for users of CI with 1 to 10 years of implant experience (mean = 4 years) and by Plath et al. [25] for users of CI after one year. Compared to the results in Rasmussen et al. [21], the baseline NCIQ total scores were approximately the same (264 points in Rasmussen et al. and 292.6 points in the current study) and then a bit higher at one year after baseline (377 points in Rasmussen et al. and 420.3 points in the current study). The same tendency can be observed when comparing current results with the NCIQ results in Häußler et al. [23] after one and two years of CI use. The SSQ-12 results in this study should be interpreted with caution, as no baseline data were available, and this measure had the highest rate of missing responses. The SSQ-12 results showed statistically significant improvements from 1 year and 1.5 years to 2 years. However, the actual mean improvements were modest and may fall below a clinically meaningful threshold. Also, the lower limit of the 95% confidence intervals approaches zero, indicating that the true effect size could be absent. This, combined with the small raw score gains, underscores the need for careful judgment about the clinical relevance of the SSQ-12 results.

A limitation of the self-reported measures overall was the response burden. The participants answered a total of 97 items at each time point across the NCIQ (60 items), SSQ-12 (12 items), and THI (25 items). In future studies, reducing the number of items may help maintain participant engagement and reduce the number of missing answers. This underscores the importance of continuously translating and culturally adapting concise, validated instruments that are suitable for longitudinal outcome assessment in a clinical setting.

### 4.4. Tinnitus Results

In addition to the findings on speech reception, self-reported hearing ability and hearing-related quality of life, change in tinnitus following cochlear implantation was also examined. Most participants—the percentage ranging from 50.0% at baseline to 78.3–82.7% throughout the study—had no or slight tinnitus. However, individual data points revealed an increase in tinnitus for some participants over time. This was also evident for participants with pre-lingual deafness and SSD. This highlights the importance of monitoring and managing tinnitus symptoms in CI recipients in the long term, both in clinical practice and in CI outcome research.

### 4.5. Additional Limitations

Other known complications following cochlear implantation include dizziness and changes in taste recognition, which were not systematically assessed in this study. Future outcome studies should—in addition to measures of efficacy, effectiveness, and change in tinnitus—include a structured assessment of these symptoms to track changes before and after implantation.

## 5. Conclusions

This prospective, longitudinal study examined speech recognition, self-reported hearing ability, hearing-related quality of life, and tinnitus severity over two years in Danish adult users of cochlear implant, with results presented separately for users of CI with post-lingual deafness, pre-lingual deafness, and single-sided deafness (SSD).

The larger cohort—users with post-lingual deafness—showed statistically significant and clinically meaningful improvements from baseline to 1, 1.5, and 2 years in both speech perception measures and in self-reported hearing-related quality of life measures. The largest gains occurred within the first post-implant year, with scores remaining stable thereafter. Ceiling effects were evident in quiet-condition speech tests after baseline, while floor effects were present for the adaptive HINT at baseline, underscoring the importance of selecting outcome measures with sufficient sensitivity across a range of hearing abilities. Among measures of speech perception in noise, the adaptive HINT detected subtle, significant improvements between one and two years that were not observed in the fixed-SNR Dantale test, supporting its value for detecting long-term changes. Self-reported NCIQ scores improved substantially from baseline to all time points after baseline, reflecting perceived benefits across physical, psychological, and social domains. SSQ-12 scores, available only after baseline, showed modest gains between 1 year (and 1.5 years) and 2 years, although these changes may fall below thresholds for clinical relevance. Tinnitus severity, as measured by the THI, remained largely stable at the group level, but individual trajectories varied, underscoring the need for ongoing monitoring during long-term clinical follow-up.

Key limitations include the over-representation of users of CI with post-lingual deafness, unbalanced hearing technology subgroups, and the absence of baseline SSQ-12 data, as well as the response burden associated with the self-reported measures. Future research should aim to include more diverse CI populations, reduce the burden of patient-reported outcome measures, and develop speech test materials sensitive to long-term change in hearing ability after cochlear implantation.

## Figures and Tables

**Table 1:** Study timeline. HINT = the adaptive Hearing in Noise Test. NCIQ = the Nijmegen Cochlear Implant Questionnaire. THI = the Tinnitus Handicap Inventory questionnaire. SSQ-12 = the 12-item Speech, Spatial, and Qualities of Hearing.

1 Year Post-CI	1.5 Years Post-CI	2 Years Post-CI
Inclusion and outcome measures: Dantale, HINT, NCIQ, SSQ-12, and THI.	Outcome measures: Dantale, HINT, NCIQ, SSQ-12, and THI.	Outcome measures: Dantale, HINT, NCIQ, SSQ-12, and THI.
Registration of hearing history and baseline outcome measures: Dantale, HINT, NCIQ, and THI.		

**Table 2:** Demographic characteristics of participants. N = number, SD = standard deviation, PTA6 = pure tone average across six frequencies, dB HL = decibel hearing level.

Demographic Characteristic	Value
Gender (N = 50)	
Female	25 (50%)
Male	25 (50%)
Age (N = 50)	
Range, years	37–83
Mean (SD), years	66.8 (10.7)
Hearing history (N = 50)	
Pre-lingual deafness	2 (4%)
Post-lingual deafness	46 (92%)
Single-sided deafness	2 (4%)
Years of experienced functional deafness (N = 46)	
Range, years	1–20
Mean (SD), years	3.8 (3.2)
Pre-implant hearing loss in CI ear (PTA6, N = 46)	
Range, dB HL	65.8–116.7
Mean (SD), dB HL	89.0 (14.5)
Pre-implant hearing loss in contralateral ear (PTA6, N = 45)	
Range, dB HL	29.2–116.7
Mean (SD), dB HL	77.6 (16.7)

**Table 3:** Post-hoc pairwise comparisons across time points for Dantale with visual cues and without visual cues. Estimates represent mean differences with standard errors (SEs), 95% confidence intervals (CIs), and Holm-adjusted *p*-values; *** *p* < 0.001. Cohen’s $d_{z}$ effect sizes are standardised using the model residual standard deviation (11.32 for Dantale with visual cues; 11.70 for Dantale without visual cues). Corresponding 95% confidence intervals are reported for each effect size.

Dantale with Visual Cues		
**Comparison**	**Estimate (SE) [95% CI],** ***p*****-Value**	$d_{z}$ **[95% CI]**
Baseline to 1 year	19.8 (2.36) [15.2, 24.4], *p* < 0.001 ***	1.75 [1.30, 2.21]
Baseline to 1.5 years	21.5 (2.44) [16.7, 26.3], *p* < 0.001 ***	1.90 [1.42, 2.37]
Baseline to 2 years	20.7 (2.50) [15.8, 25.6], *p* < 0.001 ***	1.83 [1.35, 2.31]
1 year to 1.5 years	1.7 (2.44) [−3.1, 6.5], *p* = 1.000	0.15 [−0.23, 0.63]
1 year to 2 years	0.9 (2.50) [−4.0, 5.8], *p* = 1.000	0.07 [−0.36, 0.51]
1.5 years to 2 years	0.8 (2.56) [−4.2, 5.8], *p* = 1.000	0.07 [−0.41, 0.49]
**Dantale Without Visual Cues**		
**Comparison**	**Estimate (SE) [95% CI],** ***p*****-Value**	$d_{z}$ **[95% CI]**
Baseline to 1 year	30.3 (2.44) [25.5, 35.1], *p* < 0.001 ***	2.59 [2.09, 3.09]
Baseline to 1.5 years	32.7 (2.53) [27.7, 37.7], *p* < 0.001 ***	2.79 [2.27, 3.32]
Baseline to 2 years	33.1 (2.59) [28.0, 38.2], *p* < 0.001 ***	2.83 [2.30, 3.37]
1 year to 1.5 years	2.4 (2.53) [−2.5, 7.3], *p* = 0.849	0.20 [−0.63, 0.23]
1 year to 2 years	2.8 (2.59) [−2.3, 7.9], *p* = 0.849	0.24 [−0.68, 0.20]
1.5 years to 2 years	0.4 (2.65) [−4.8, 5.6], *p* = 0.870	0.04 [−0.49, 0.41]

**Table 4:** Post-hoc pairwise comparisons across time points for the Dantale with background noise and the adaptive HINT tests. Estimates represent mean differences with standard errors (SEs), 95% confidence intervals (CIs), and Holm-adjusted *p*-values; * *p* < 0.05, *** *p* < 0.001. Cohen’s $d_{z}$ effect sizes are standardised using the model residual standard deviation (12.00 for Dantale with background noise; 1.86 for the adaptive HINT test). The 95% confidence intervals are reported for the effect sizes.

Dantale with Background Noise		
**Comparison**	**Estimate (SE) [95% CI],** ***p*****-Value**	$d_{z}$ **[95% CI]**
Baseline to 1 year	32.3 (2.50) [27.4, 37.2], *p* < 0.001 ***	2.69 [2.18, 3.20]
Baseline to 1.5 years	36.9 (2.60) [31.8, 42.0], *p* < 0.001 ***	3.07 [2.53, 3.62]
Baseline to 2 years	37.6 (2.67) [32.4, 42.8], *p* < 0.001 ***	3.13 [2.57, 3.69]
1 year to 1.5 years	4.6 (2.60) [−0.5, 9.7], *p* = 0.160	0.38 [−0.05, 0.81]
1 year to 2 years	5.3 (2.67) [0.0, 10.6], *p* = 0.145	0.44 [0.00, 0.89]
1.5 years to 2 years	0.7 (2.72) [−4.6, 6.0], *p* = 0.790	0.06 [−0.39, 0.51]
**Adaptive HINT**		
**Comparison**	**Estimate (SE) [95% CI],** ***p*****-Value**	$d_{z}$ **[95% CI]**
Baseline to 1 year	−4.7 (0.59) [−5.9, −3.6], *p* < 0.001 ***	2.50 [1.79, 3.21]
Baseline to 1.5 years	−5.1 (0.61) [−6.3, −3.9], *p* < 0.001 ***	2.72 [1.98, 3.46]
Baseline to 2 years	−5.8 (0.63) [−7.0, −4.6], *p* < 0.001 ***	3.10 [2.32, 3.87]
1 year to 1.5 years	−0.4 (0.44) [−1.3, 0.5], *p* = 0.349	0.22 [−0.25, 0.69]
1 year to 2 years	−1.1 (0.45) [−2.0, −0.1], *p* = 0.042 *	0.60 [0.12, 1.09]
1.5 years to 2 years	−0.7 (0.45) [−1.6, 0.2], *p* = 0.236	0.38 [−0.10, 0.86]

**Table 5:** Summary statistics for all measures at each time point for users of CI with post-lingual deafness. Group scores are presented as group means with associated standard deviations (SDs).

Measure, Unit	Baseline	1 Year	1.5 Years	2 Years
Dantale with visual cues, %	74.2 (21.9)	94.0 (6.8)	95.7 (3.8)	94.9 (4.1)
Dantale without visual cues, %	58.9 (23.5)	89.3 (9.3)	91.7 (6.4)	92.1 (5.9)
Dantale with background noise, %	23.4 (20.9)	55.7 (15.5)	60.2 (13.5)	61.7 (11.2)
Adaptive HINT, dB SNR	8.6 (4.2)	4.9 (3.1)	4.8 (2.9)	4.2 (2.7)
SSQ-12, points	—	4.7 (1.6)	4.7 (1.5)	5.1 (1.7)
NCIQ, points	292.6 (83.6)	420.3 (54.5)	430.8 (74.3)	436.3 (67.1)
THI, points	9.7 (15.3)	8.4 (14.3)	6.2 (10.0)	7.9 (13.6)

**Table 6:** Post-hoc pairwise comparisons across time points for SSQ-12 and NCIQ. Estimates represent mean differences (SEs), 95% confidence intervals (CIs), and Holm-adjusted *p*-values; * *p* < 0.05, *** *p* < 0.001. Cohen’s $d_{z}$ is standardised by the model residual SD (0.70 for SSQ-12; 46.77 for NCIQ); 95% confidence intervals are shown.

SSQ-12		
**Comparison**	**Estimate (SE) [95% CI],** ***p*****-Value**	$d_{z}$ **[95% CI]**
Baseline to 1 year	—	—
Baseline to 1.5 years	—	—
Baseline to 2 years	—	—
1 year to 1.5 years	0.11 (0.21) [−0.30, 0.52], *p* = 0.586	0.16 [−0.43, 0.75]
1 year to 2 years	0.60 (0.21) [0.18, 1.02], *p* = 0.015 *	0.86 [0.03, 1.05]
1.5 years to 2 years	0.49 (0.20) [0.09, 0.89], *p* = 0.042 *	0.70 [0.10, 1.29]
**NCIQ**		
**Comparison**	**Estimate (SE) [95% CI],** ***p*****-Value**	$d_{z}$ **[95% CI]**
Baseline to 1 year	130.5 (11.1) [108.7, 152.3], *p* < 0.001 ***	2.79 [2.22, 3.36]
Baseline to 1.5 years	139.7 (11.5) [117.2, 162.2], *p* < 0.001 ***	2.99 [2.39, 3.58]
Baseline to 2 years	145.3 (11.5) [122.8, 167.8], *p* < 0.001 ***	3.11 [2.50, 3.71]
1 year to 1.5 years	9.12 (10.2) [−11.0, 29.2], *p* = 0.751	0.20 [−0.24, 0.63]
1 year to 2 years	14.71 (10.3) [−5.4, 34.9], *p* = 0.473	0.32 [−0.13, 0.76]
1.5 years to 2 years	5.59 (10.6) [−15.1, 26.3], *p* = 0.751	0.12 [−0.33, 0.57]

**Table 7:** The percentage of users of CI with post-lingual deafness in each THI category at each time point.

THI Category	Baseline	1 Year	1.5 Years	2 Years
No tinnitus	32.6%	45.7%	47.8%	50.0%
Slight	17.4%	37.0%	32.6%	28.3%
Mild	10.9%	13.0%	2.2%	6.5%
Moderate	2.2%	2.2%	4.3%	4.3%
Severe	2.2%	0.0%	0.0%	2.2%
Catastrophic	0.0%	2.2%	0.0%	0.0%

## Data Availability

Data are not publicly available due to privacy restrictions, as participants did not provide consent for their data to be shared beyond the research team directly involved in this study.

## Appendix A. Observed and Missing Data

Table A1 summarises the number of observed and missing data points across all measures at each time point for users of CI with post-lingual deafness.

**Table A1:** Observed and missing data by measure and time point for users of CI with post-lingual deafness (Total N = 46 per test). NCIQ = the Nijmegen Cochlear Implant Questionnaire. SSQ-12 = the 12-item Speech, Spatial, and Qualities of Hearing. THI = the Tinnitus Handicap Inventory questionnaire.

Measure	Time Point	Observed	Missing	Missing (%)
Dantale with visual cues	Baseline	46	0	0.0%
	1 year	46	0	0.0%
	1.5 years	41	5	10.9%
	2 years	38	8	17.4%
Dantale without visual cues	Baseline	46	0	0.0%
	1 year	46	0	0.0%
	1.5 years	41	5	10.9%
	2 years	38	8	17.4%
Dantale with background noise	Baseline	46	0	0.0%
	1 year	46	0	0.0%
	1.5 years	41	5	10.9%
	2 years	38	8	17.4%
Adaptive HINT	Baseline	16	30	65.2%
	1 year	41	5	10.9%
	1.5 years	37	9	19.6%
	2 years	35	11	23.9%
NCIQ	Baseline	32	14	30.4%
	1 year	46	0	0.0%
	1.5 years	40	6	13.0%
	2 years	39	7	15.2%
SSQ-12	1 year	31	15	32.6%
	1.5 years	26	20	43.5%
	2 years	28	18	39.1%
THI	Baseline	30	16	34.8%
	1 year	46	0	0.0%
	1.5 years	40	6	13.0%
	2 years	42	4	8.7%

## References

[1] (2025). World Health Organization.Deafness and Hearing Loss. https://www.who.int/news-room/fact-sheets/detail/deafness-and-hearing-loss.

[2] Andries E., Gilles A., Topsakal V., Vanderveken O.M., Van de Heyning P., Van Rompaey V., Mertens G. (2021). Systematic Review of Quality of Life Assessments after Cochlear Implantation in Older Adults. Audiol. Neurotol..

[3] Lin F.R., Yaffe K., Xia J., Xue Q.L., Harris T.B., Purchase-Helzner E., Satterfield S., Ayonayon H.N., Ferrucci L., Simonsick E.M. (2013). Hearing Loss and Cognitive Decline in Older Adults. JAMA Intern. Med..

[4] Sarant J.Z., Busby P.A., Schembri A.J., Briggs R.J.S., Masters C.L., Harris D.C. (2024). COCHLEA: Longitudinal Cognitive Performance of Older Adults with Hearing Loss and Cochlear Implants at 4.5-Year Follow-Up. Brain Sci..

[5] Sarant J., Harris D., Busby P., Maruff P., Schembri A., Lemke U., Launer S. (2020). The effect of hearing aid use on cognition in older adults: Can we delay decline or even improve cognitive function?. J. Clin. Med..

[6] Babajanian E.E., Gurgel R.K. (2022). Cognitive and behavioral effects of hearing loss. Curr. Opin. Otolaryngol. Head Neck Surg..

[7] Boisvert I., Reis M., Au A., Cowan R., Dowell R.C. (2020). Cochlear implantation outcomes in adults: A scoping review. PLoS ONE.

[8] Bond M., Mealing S., Anderson R., Elston J., Weiner G., Taylor R.S., Hoyle M., Liu Z., Price A., Stein K. (2009). The effectiveness and cost-effectiveness of cochlear implants for severe to profound deafness in children and adults: A systematic review and economic model. Health Technol. Assess..

[9] Yang Z., Cosetti M. (2016). Safety and outcomes of cochlear implantation in the elderly: A review of recent literature. J. Otol..

[10] McRackan T.R., Bauschard M., Hatch J.L., Franko-Tobin E., Droghini H.R., Nguyen S.A., Dubno J.R. (2018). Meta-analysis of quality-of-life improvement after cochlear implantation and associations with speech recognition abilities. Laryngoscope.

[11] Tang D., Tran Y., Lo C., Lee J.N., Turner J., McAlpine D., McMahon C., Gopinath B. (2024). The Benefits of Cochlear Implantation for Adults: A Systematic Umbrella Review. Ear Hear..

[12] Basura G., Cienkowski K., Hamlin L., Ray C., Rutherford C., Stamper G., Schooling T., Ambrose J. (2023). American Speech-Language-Hearing Association Clinical Practice Guideline on Aural Rehabilitation for Adults with Hearing Loss. Am. J. Audiol..

[13] Cusumano C., Friedmann D.R., Fang Y., Wang B., Roland J.T., Waltzman S.B. (2017). Performance Plateau in Prelingually and Postlingually Deafened Adult Cochlear Implant Recipients. Otol. Neurotol..

[14] Dunn C.C., Zwolan T.A., Balkany T.J., Strader H.L., Biever A., Gifford R.H., Hall M.W., Holcomb M.A., Hill H., King E.R. (2024). A Consensus to Revise the Minimum Speech Test Battery—Version 3. Am. J. Audiol..

[15] Gifford R.H., Shallop J.K., Peterson A.M. (2008). Speech Recognition Materials and Ceiling Effects: Considerations for Cochlear Implant Programs. Audiol. Neurotol..

[16] Lenarz M., Sönmez H., Joseph G., Büchner A., Lenarz T. (2012). Long-Term Performance of Cochlear Implants in Postlingually Deafened Adults. Otolaryngol. Head Neck Surg..

[17] Billings C.J., Olsen T.M., Charney L., Madsen B.M., Holmes C.E. (2024). Speech-in-Noise Testing: An Introduction for Audiologists. Semin. Hear..

[18] Mertens G., Andries E., Clement C., Cochet E., Hofkens Van den Brandt A., Jacquemin L., Joossen I., Vermeersch H., Lammers M.J.W., Van Rompaey V. (2024). Contralateral hearing aid use in adult cochlear implant recipients: Retrospective analysis of auditory outcomes. Int. J. Audiol..

[19] Távora-Vieira D., Wedekind A., Acharya A., Kuthubutheen J., Voola M., Cavalheri V., Friedland P. (2025). Advanced age is not a predictor for cochlear implantation outcomes in adults with moderate to profound sensorineural hearing loss. Braz. J. Otorhinolaryngol..

[20] Kelsall D., Lupo J., Biever A. (2021). Longitudinal outcomes of cochlear implantation and bimodal hearing in a large group of adults: A multicenter clinical study. Am. J. Otolaryngol..

[21] Rasmussen K.M.B., West N.C., Bille M., Sandvej M.G., Cayé-Thomasen P. (2022). Cochlear Implantation Improves Both Speech Perception and Patient-Reported Outcomes: A Prospective Follow-Up Study of Treatment Benefits among Adult Cochlear Implant Recipients. J. Clin. Med..

[22] Buchman C.A., Herzog J.A., McJunkin J.L., Wick C.C., Durakovic N., Firszt J.B., Kallogjeri D. (2020). Assessment of Speech Understanding After Cochlear Implantation in Adult Hearing Aid Users: A Nonrandomized Controlled Trial. JAMA Otolaryngol. Head Neck Surg..

[23] Häußler S.M., Knopke S., Wiltner P., Ketterer M., Gräbel S., Olze H. (2019). Long-term Benefit of Unilateral Cochlear Implantation on Quality of Life and Speech Perception in Bilaterally Deafened Patients. Otol. Neurotol..

[24] Hirschfelder A., Gräbel S., Olze H. (2008). The impact of cochlear implantation on quality of life: The role of audiologic performance and variables. Otolaryngol. Head Neck Surg..

[25] Plath M., Marienfeld T., Sand M., van de Weyer P.S., Praetorius M., Plinkert P.K., Baumann I., Zaoui K. (2022). Prospective study on health-related quality of life in patients before and after cochlear implantation. Eur. Arch. Otorhinolaryngol..

[26] Ma C., Fried J., Nguyen S.A., Schvartz-Leyzac K.C., Camposeo E.L., Meyer T.A., Dubno J.R., McRackan T.R. (2023). Longitudinal Speech Recognition Changes After Cochlear Implant: Systematic Review and Meta-analysis. Laryngoscope.

[27] Elberling C., Ludvigsen C., Lyregaard P.E. (1989). Dantale: A New Danish Speech Material. Scand. Audiol..

[28] Nielsen J.B., Dau T. (2011). The Danish Hearing in Noise Test. Int. J. Audiol..

[29] Hinderink J.B., Krabbe P.F., van den Broek P. (2000). Development and application of a health-related quality-of-life instrument for adults with cochlear implants: The Nijmegen Cochlear Implant Questionnaire. Otolaryngol. Head Neck Surg..

[30] Neumann C.S., Schmidt J.H. (2025). Evaluation of the Nijmegen Cochlear Implant Questionnaire in Danish. Int. Arch. Otorhinolaryngol..

[31] Noble W., Jensen N.S., Naylor G., Bhullar N., Akeroyd M.A. (2013). A short form of the Speech, Spatial and Qualities of Hearing scale suitable for clinical use: The SSQ12. Int. J. Audiol..

[32] Newman C.W., Jacobson G.P., Spitzer J.B. (1996). Development of the Tinnitus Handicap Inventory. Arch. Otolaryngol. Head Neck Surg..

[33] Casarella A., Notaro A., Laria C., Serra N., Genovese E., Malesci R., Auletta G., Fetoni A.R. (2024). State-of-the-Art on the Impact of Bimodal Acoustic Stimulation on Speech Perception in Noise in Adults: A Systematic Review. Audiol. Res..

[34] Sedaghat A.R. (2019). Understanding the Minimal Clinically Important Difference (MCID) of Patient-Reported Outcome Measures. Otolaryngol. Head Neck Surg..

